# Nanoscale Effect of Zirconia Filler Surface on Mechanical Tensile Strength of Polymer Composites

**DOI:** 10.1186/s11671-020-3282-6

**Published:** 2020-03-02

**Authors:** Kai Kan, Daiki Moritoh, Yuri Matsumoto, Kanami Masuda, Masataka Ohtani, Kazuya Kobiro

**Affiliations:** 1 School of Environmental Science and Engineering, Kochi University of Technology, 185 Miyanokuchi, Tosayamada, Kochi 782-8502 Japan; 2 Laboratory for Structural Nanochemistry, Kochi University of Technology, 185 Miyanokuchi, Tosayamada, Kochi 782-8502 Japan; 3 Research Center for Material Science and Engineering, Kochi University of Technology, 185 Miyanokuchi, Tosayamada, Kochi 782-8502 Japan

**Keywords:** Nanoparticle, Nano-concave-convex, Impregnation, Surface modification, Polymer composite

## Abstract

A characteristic effect of a nano-concave-convex structure of a zirconia nanoparticle assembly with an inherent porous structure and huge surface area enabled us to introduce systematic surface modification by thermal treatment to smooth surface and polymer impregnation to mask the nano-concave-convex structure of the zirconia nanoparticle assembly. A polymer composite prepared from 30 wt% poly(*N*-isopropylacrylamide) containing 0.02 wt% zirconia nanoparticle assembly with the inherent nano-concave-convex surface structure showed the highest tensile strength in mechanical tensile testing. However, both sintered zirconia nanoparticle assembly with smooth surface and zirconia nanoparticle assemblies with polymer masked surface showed lower strength with longer elongation at break in mechanical tensile testing.

## Introduction

Nanomaterials are some of the most intriguing advanced materials in many research and application fields [1–5], since their intrinsic physical/chemical properties are so different from those of bulk materials [6–9]. When nanomaterials are applied as fillers of polymer composites, minute differences in the fillers, such as surface area, surface structure, and particle morphology of the nanomaterials, lead to drastic changes in the macro-scale properties of the composites [10].

For instance, incorporation of inorganic components into polymers improved physical and chemical properties, such as thermal stability, mechanical strength, dispersibility, and solubility [11–14].

However, a systematic study failed to clarify the relationship between the nanoscale properties of the nanomaterials, i.e., structure, morphology, and surface area, and the macro-scale physical and mechanical ones of the composites.

Porous metal oxide nanomaterials such as silicon dioxide [15–17], titanium dioxide [18–20], zirconium dioxide [21–23], cerium dioxide [24–26], and other materials [27–29] with large surface areas have been applied in chemical catalysis, gas absorption, separation, drug delivery, and energy storage materials [30–35]. In this context, our group developed unique metal oxide nanoparticle assemblies with submicron-sized spherical morphologies by a simple one-pot and single-step solvothermal approach [36]. We named these materials *m*icro/*m*esoporously *a*rchitected *r*oundly *i*ntegrated *m*etal *o*xides (MARIMOs). These produce nanometer-scale surface roughness and wide surface area. For instance, TiO_2_ MARIMO has a nano-concave-convex surface due to its inherent numerous fine primary particles with diameters of ca. 5 nm and high specific surface area (400 m^2^ g^−1^) [35]. In previous research, we applied these unique materials to support inhomogeneous nanometal catalysts and anode materials for rechargeable batteries. In the catalyst supports, highly dispersed Au nanoparticles on the TiO_2_ MARIMO support surface enhanced the catalytic activity and improved the durability of the catalyst at high temperature [37]. In the anode material, Nb_2_O_5_-TiO_2_ MARIMO increased the current capacity and lifetime of the batteries [38]. Additionally, TiO_2_ nanofiber bundles with a cheek brush morphology enhanced the mechanical strength of a polymer hydrogel when used as a filler [39].

**Fig. 1:**
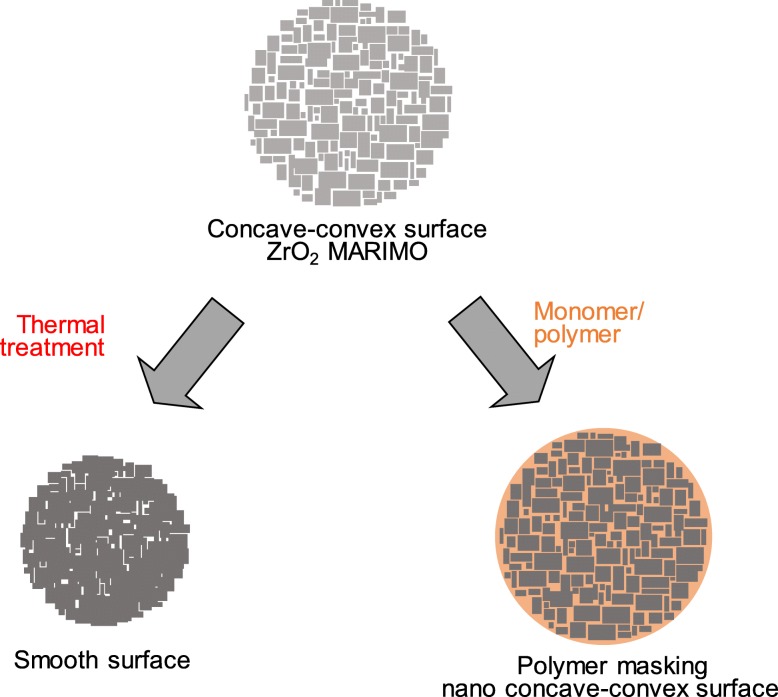
Schematic of surface modification of ZrO_2_ MARIMO by thermal treatment and impregnation with monomer/polymer

The nano- and micro-scale anchoring effect is most significant in the adhesive mechanism. We consider that these MARIMOs with nano-concave-convex surface structures, huge surface areas, and porous structures would be appropriate for clarifying the relationship between nanoscale surface properties and macro-scale material properties, since the surface properties of MARIMO can be easily tuned by thermal treatment and polymer decoration (Fig. 1). For instance, thermal treatment of MARIMO creates a smooth surface with a decreased surface area and lower porosity. Impregnation [40] of monomers or polymers into MARIMO pores should mask the nano-concave-convex surface of the MARIMO. Thus, in this paper, a new method of filler surface modification by impregnation of polymers to mask the nano-concave-convex shape of MARIMO was studied to demonstrate the nano-anchoring effect of the filler surface. Here, we selected a zirconia (ZrO_2_) MARIMO as a filler to enhance the mechanical properties of polymer composites, since ZrO_2_ filler exhibits better properties such as chemical resistance especially for acids, mechanical strength, and thermal stability, which would be favorable for the polymeric matrix to lead durable polymer composites [41–43]. Monomers, 2-hydroxyethyl methacrylate (HEMA), benzyl methacrylate (BMA), and cyclohexyl methacrylate (CHMA), and their polymers were selected to modify the nano-concave-convex surface of ZrO_2_ MARIMO fillers. Poly(*N*-isopropylacrylamide) (PNIPAM) hydrogel was chosen as a matrix for the polymer composites.

There are several approaches to estimate physical and chemical interactions between filler surfaces and polymer chains in polymer composites. Thermogravimetry, UV–visible spectroscopy, FT-IR spectroscopy, and microscopy are their representatives. Here, we adopted mechanical tensile testing as an alternative technique that is relatively simple, easy, and rapid. There are a few reports on mechanical property of the hydrogels with graphene oxide and ZrO_2_ powder [44, 45], which is different from ours with respect to a simple system which consisted of only zirconia and polymer matrix. To the best of our knowledge, no report on the relationship between nanostructural changes in filler surfaces and polymer chains in polymer composites has been published.

## Methods

### Materials

*N*-isopropylacrylamide (NIPAM), *N,N,N',N'*-tetramethylethylenediamine (TMEDA), potassium persulfate (KPS), and commercial zirconia (commercial ZrO_2_) were purchased from FUJIFILM Wako Pure Chemical Corporation. HEMA, BMA, CHMA, and 1-hydroxycyclohexyl phenyl ketone (HCPK) were purchased from Tokyo Chemical Industry Co., Ltd. All reagents were used as received. ZrO_2_ MARIMO was obtained from UJIDEN Chemical Industry Co., Ltd.

### Preparation of HEMA-, NIPAM-, BMA-, and CHMA-Impregnated ZrO_2_ MARIMO Fillers

An impregnation method for supported nanometal catalyst preparation [40] was applied to obtain HEMA-impregnated ZrO_2_ MARIMO filler. ZrO_2_ MARIMO was dried at 80 °C under vacuum for 12 h. Then, 20 μL of a HEMA/HCPK (20/1, mol/mol) mixture was added to 200 mg of vacuum-dried ZrO_2_ MARIMO, and the mixture was manually mixed well with a mortar and pestle. It was then irradiated with UV light for 1 h with intermittent mixing every 15 min. Similar procedures were used to prepare NIPAM-, BMA-, and CHMA-impregnated ZrO_2_ MARIMO fillers.

### Preparation of PNIPAM Hydrogels with ZrO_2_ Fillers

Hydrogels consisting of PNIPAM and ZrO_2_ fillers were prepared according to a previously reported method [37]. ZrO_2_ MARIMO (24 mg, 0.02 wt%) was dispersed in 115 mL of reverse osmosis water with N_2_ bubbling before adding NIPAM (36 g, 30 wt%) to the solution. The mixture was stirred for 30 min and then KPS (0.18 g, 0.67 mmol) in water (5 mL) and TMEDA (1.8 mL, 12 mmol) were successively added. The mixture was carefully transferred to several glass tubes with an inner diameter of 1.0 cm. The upper dead volume of the tubes was purged with N_2_ and the glass tubes were closed tightly with screw caps then left at 25 °C. After 3 days, the glass tubes were cut and the polymer hydrogels were removed. The obtained hydrogel rods with a diameter of 1.0 cm and length of 3.0 cm were used for mechanical strength measurements. Similar procedures afforded PNIPAM composites with HEMA-, NIPAM-, BMA-, and CHMA-impregnated ZrO_2_ MARIMO fillers.

### Mechanical Tensile Testing of Polymer Composites

Mechanical tensile testing was applied to the specimens in the axial direction. The deformed length of the composite (strain) and the applied force (stress) were measured using a tensile tester (AND MCT-2150**)** with a crosshead speed of 50 mm min^−1^ at room temperature. On the tensile tests of all composite samples, the percent elongation, 930%, is the limitation of the tensile tester machine. Ten composite samples were used for the mechanical tensile testing, and at least seven samples were used for data analysis. We note that composites containing bubble, cracked by glass, and cracked by grip of tensile testing machine were omitted from the data analysis to guarantee the data quality. The results are presented as mean ± standard deviation.

### Characterization Methods

Scanning electron microscopy (SEM) was performed on a Hitachi SU8020 FE-SEM microscope. Transmission electron microscopy (TEM) images were obtained with a JEOL JEM-2100F microscope. STEM-EDX mapping was taken by bright-field (BF) mode and performed on an Oxford INCA X-max 80 EDX spectrometer. X-ray diffractometry (XRD) was performed on a Rigaku SmartLab diffractometer (Cu Kα radiation, D/teX Ultra 250 detector). Nitrogen adsorption–desorption isotherms were obtained using a BEL Japan Inc. Belsorp Mini (II) instrument. Specific surface areas were calculated using the Brunauer–Emmett–Teller (BET) method, and pore size distribution was derived by the Barrett–Joyner–Halenda (BJH) method. Differential scanning calorimetry (DSC) was performed with DSC7000X from Hitachi High-Tech Science Corporation at a scanning rate of 10 °C min^-1^ from 0 to 100 °C in a nitrogen atmosphere in three scans. Size exclusion chromatography (SEC) was performed using a JASCO PU-2080 Plus pump with two gel columns (KF-804L and KF-806L) and an RI-2031 Plus Intelligent RI detector in chloroform calibrated with polystyrene standards at 40 °C. Diffuse reflectance infrared Fourier transform spectroscopy (DRIFTS) was performed on a FT/IR-4600 from JASCO Corporation, and the spectra were performing Kubelka-Munk transformations.

## Results and Discussion

### Surface Properties of ZrO_2_ MARIMOs

**Fig. 2:**
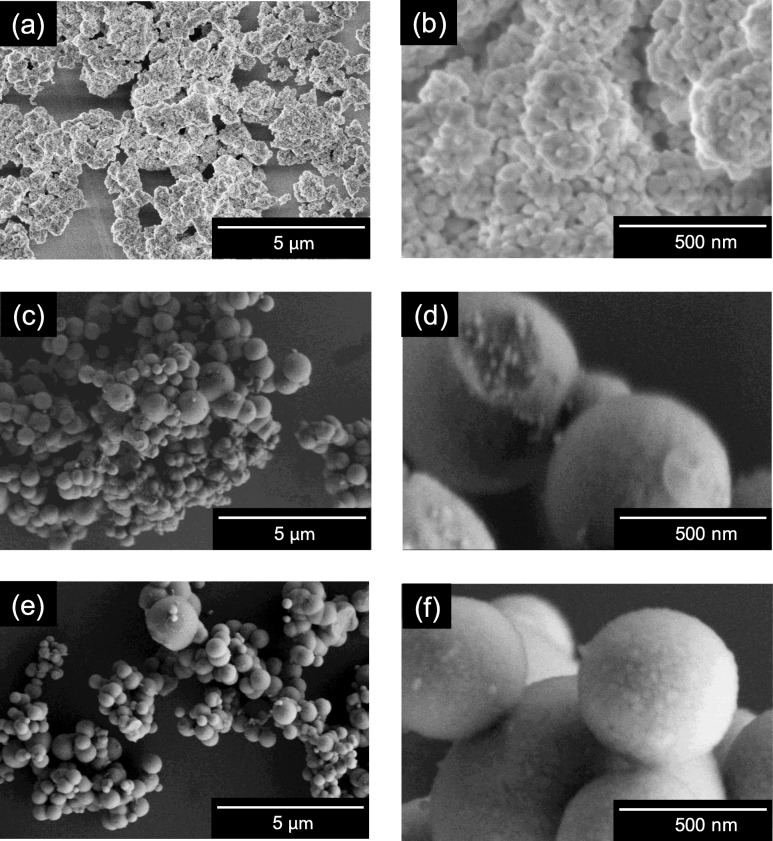
SEM images of **a** low magnification and **b** high magnification of commercially available ZrO_2_, **c** low magnification and **d** high magnification of ZrO_2_ MARIMO, and **e** low magnification and **f** high magnification of sintered ZrO_2_ MARIMO

Physical properties of ZrO_2_ fillers were evaluated by the BET method, XRD, and SEM. A reference material, commercial ZrO_2_ nanoparticles, showed roughly aggregated morphology (Fig. 2a,b) with a specific surface area of 20 m^2^ g^−1^ (Table 1). Conversely, ZrO_2_ MARIMO exhibited a spherical mesoporous morphology (Fig. 2c,d) with a huge specific surface area (283 m^2^ g^−1^), which is 14 times larger than that of the commercial ZrO_2_ nanoparticles. Sintered ZrO_2_ MARIMO obtained by heating at 700 °C for 3 h in air exhibited a low specific surface area of 6 m^2^ g^−1^ as expected (Fig. 2e,f). The extreme reduction in the specific surface area indicates that the primary particle size of the sintered ZrO_2_ MARIMO was increased by heating, which was confirmed by primary particle size estimation from the XRD peak width using the Scherrer equation and BJH method from the nitrogen adsorption–desorption isotherm analysis (Table 1). Thus, tiny primary particles in the ZrO_2_ MARIMO brought huge surface area as well as porous structure with the nano-concave-convex surface. Therefore, much amount of materials will interact with the nano-concave-convex surface and pores of the ZrO_2_ MARIMO.

**Table 1:** Specific surface area and primary particle size of ZrO_2_ fillers

Filler	Specific surface are^a^ (m^2^ g^-1^)	Pore size^b^ (nm)	Primary particle size^c^ (nm)
Commercial ZrO_2_	20	33	31
ZrO_2_ MARIMO	283	2	1
Sintered ZrO_2_ MARIMO	6	10	20

^a^Determined by BET method

^b^Determined by BJH method

^c^Estimated by the Scherrer equation

DSC is a powerful tool to demonstrate the interaction between ZrO_2_ filler surface and organic molecules through endo- or exo-thermal phenomena [45]. Prior to surface decollation of ZrO_2_ MARIMO fillers by polymers, we selected NIPAM monomer as probe molecules to investigate the interaction between the ZrO_2_ filler surface and organic probe molecules by means of DSC, since melting point of NIPAM monomer is sensitive to the crystallinity of NIPAM solid [46]. If NIPAM solid is embedded in pores of host materials, its melting point would shift to lower temperature, since the crystallinity of the embedded nano-sized NIPAM solid with nano-size in the pores of the host materials would be easily disturbed by some perturbation from boundary pore wall.

The DSC profiles of NIPAM itself, a mixture of commercial ZrO_2_ nanoparticles and NIPAM monomer (commercial ZrO_2_/NIPAM), a mixture of ZrO_2_ MARIMO and NIPAM monomer (ZrO_2_ MARIMO/NIPAM), and a mixture of sintered ZrO_2_ MARIMO and NIPAM monomer (sintered ZrO_2_ MARIMO/NIPAM) with different ZrO_2_/NIPAM ratio in weight are shown in Fig. S1, Fig. S2, and Fig. S3, respectively. NIPAM monomer itself showed an endothermal peak ascribed to its melting point at 67.7 °C. In the case of commercial ZrO_2_/NIPAM, a simple gradual peak shift of DSC profiles from 67.7 °C (NIPAM) to 64.8 °C was observed in accordance with the higher ZrO_2_ contents (Fig. S1, Table S1). The endothermal peaks at 64.8 °C can be ascribed to the melting point of NIPAM solid situated between the commercial ZrO_2_ primary particles.

On the contrary, the larger temperature shifts from 67.7 to 62.4 °C were recognized in accordance with the higher ZrO_2_ MARIMO contents up to ZrO_2_ MARIMO/NIPAM = 50/50 (wt%) in the case of ZrO_2_ MARIMO/NIPAM (Fig. S2, Table S1). These large shifts clearly demonstrate that the ZrO_2_ MARIMO has some positive effect on solid state of NIPAM monomer embedded in the MARIMO pores, where the larger shift of the endothermal peaks could correspond to the stronger interaction between the ZrO_2_ filler surface and NIPAM monomer. However, the endothermal peaks were shifted to opposite direction of higher temperature of 65.2 °C at the ZrO_2_/NIPAM ratio ranging from 50/50 to 80/20 (wt%). It is difficult to put forward a conclusive discussion, but the endothermal peaks at 62.4 and 65.2 °C might be ascribed to the melting points of NIPAM solid embedded deep pores and much amount of shallow pores in ZrO_2_ MARIMO, respectively. As for the sintered ZrO_2_ MARIMO/NIPAM with the smooth surface, quite similar simple lower temperature shifts of endothermal peaks were shown in proportion to sintered ZrO_2_ MARIMO contents (Fig. S3, Table S1) similar to the results of commercial ZrO_2_/NIPAM in Fig. S1.

Thus, the positive interaction found between the ZrO_2_ MARIMO and NIPAM monomer would be advantageous to polymer decollation onto the ZrO_2_ MARIMO surface.

### Preparation of PNIPAM Hydrogels with ZrO_2_ Fillers

To evaluate the effect of surface structure of the ZrO_2_ fillers further, PNIPAM hydrogels with the ZrO_2_ fillers were chosen, since strength of PNIPAM hydrogels were sensitive to the properties of the fillers. PNIPAM hydrogels were prepared from aqueous solutions containing different amounts (20, 25, and 30 wt%) of NIPAM, KPS as a radical initiator, and TMEDA. When the gel obtained from 20 wt% of NIPAM was left at room temperature, it changed to sol within 60 min (Fig. S4a). Conversely, no structural deformation of the hydrogel shape was observed with the gels obtained from 25 and 30 wt% NIPAM solutions (Fig. S4b–c). The stress and strain were 2.7 ± 0.2 kPa and above 930% for 25 wt% PNIPAM hydrogels, and 7.8 ± 0.2 kPa and 716 ± 106% for 30 wt% PNIPAM hydrogels, respectively (Fig. S5 and Table S2). Then, we selected the stronger 30 wt% PNIPAM hydrogel as a polymer matrix to evaluate the effect of the ZrO_2_ fillers.

The ZrO_2_ filler content in PNIPAM hydrogels was then optimized by changing the amount of the commercial ZrO_2_ filler (0.002 (2a), 0.02 (2b), and 0.04 wt% (2c)) in 30 wt% of PNIPAM hydrogel. Consequently, composite 2a showed the highest tensile strength (9.5 ± 0.7 kPa), while composite 2b showed the highest elongation (902 ± 28%) among all composites (Fig. S6 and Table S3). From these results, it is difficult to judge which one (high tensile strength or long elongation) is suitable for the filler amount to prepare polymer hydrogel. Then, the profile areas were calculated to estimate how much work was necessary to break these composites. Consequently, composite 2b showed the highest work among all the composites (Table S3).

Thus, the conditions of 30 wt% of NIPAM and 0.02 wt% of ZrO_2_ filler were fixed throughout the experiments.

### Mechanical Tensile Testings of the Polymer Composites

**Fig. 3:**
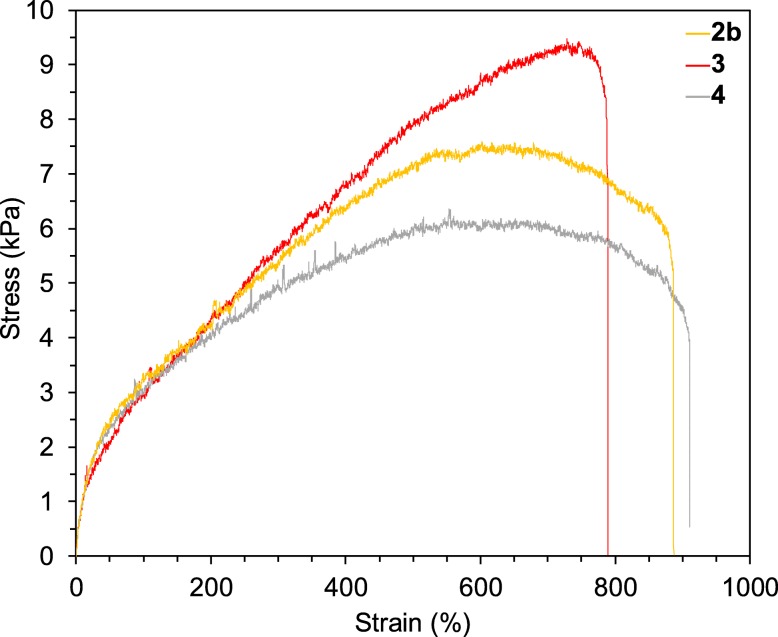
Tensile stress–strain curves for 30 wt% PNIPAM hydrogel composites with 0.02 wt% ZrO_2_ fillers. Composites with 0.02 wt% commercial ZrO_2_ (2b), with 0.02 wt% nano-concave-convex surface ZrO_2_ MARIMO (3), and with 0.02 wt% sintered ZrO_2_ MARIMO (4)

The mechanical tensile strength obtained from stress–strain curves is shown in Fig. 3. The maximum stress and strain of each composite estimated from mechanical tensile testings are summarized in Table 2. The effect of the surface morphology of ZrO_2_ filler on polymer composites can be clarified by measuring the tensile strength [41–43] of composites prepared with commercial ZrO_2_ (2b), with nano-concave-convex ZrO_2_ MARIMO (3), with and sintered ZrO_2_ MARIMO (4) with the smooth surface. As a result, composite 3 containing MARIMO with the nano-concave-convex surface showed the highest ultimate tensile strength (9.2 ± 0.2 kPa) and the lowest elongation (746 ± 37%). However, composite 4 containing sintered MARIMO with a smooth surface exhibited a poor tensile strength of 6.6 ± 0.3 kPa. Elongation capacities of 2b (902 ± 28%) and 4 (903 ± 19%) were almost the same. Generally, the anchor effect of filler surface on polymer chains and slippage of polymer chains can be estimated from the maximum stress and maximum strain, respectively, obtained from mechanical tensile testing [47]. The obtained results clearly indicate that the nano-concave-convex surface played a critical role in the tensile strength of the polymer composites as expected, which can be an anchoring effect between the nano-concave-convex surface and the polymer chains in the polymer composites. In order to know the microstructure and distribution of ZrO_2_ fillers, we used freeze-dried polymer composites for direct SEM observation. As a result, we confirmed the uniform polymer network of hydrogel but no aggregation nor agglomeration of ZrO_2_ fillers was observed (Fig. S7).

**Table 2:** Tensile strengths and elongation capacities of 30 wt% PNIPAM hydrogel composites with 0.02 wt% ZrO_2_ nano assembly

Composite	Filler	Stress_MAX_ (kPa)	Strain_MAX_ (%)
1	–	7.8 ± 0.2	716 ± 106
2b	Commercial ZrO_2_	7.7 ± 0.2	902 ± 28
3	ZrO_2_ MARIMO	9.2 ± 0.2	746 ± 37
4	Sintered ZrO_2_ MARIMO	6.6 ± 0.3	903 ± 19

### Effect of Surface Properties of Polymer-Impregnated ZrO_2_ MARIMO

**Fig. 4:**
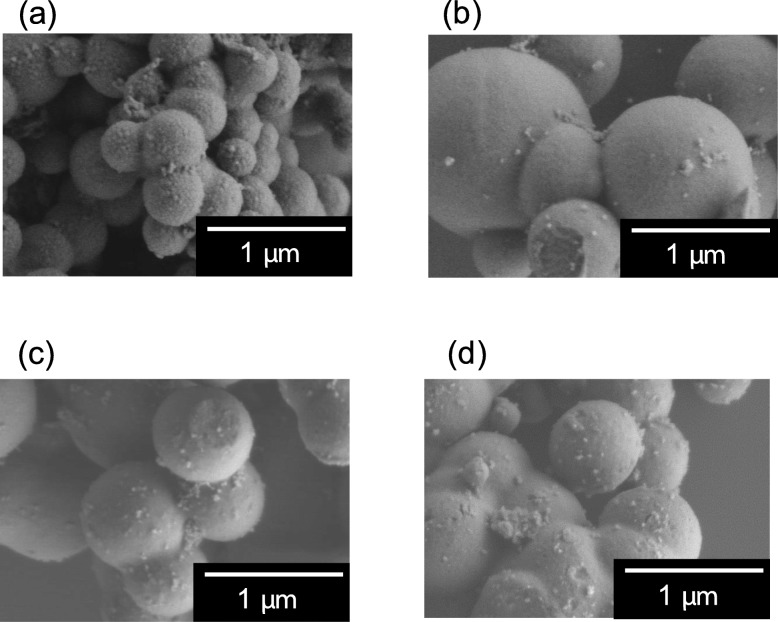
SEM images of **a** HEMA-impregnated ZrO_2_ MARIMO, **b** NIPAM-impregnated ZrO_2_ MARIMO, **c** BMA-impregnated ZrO_2_ MARIMO, and **d** CHMA-impregnated ZrO_2_ MARIMO

**Fig. 5:**
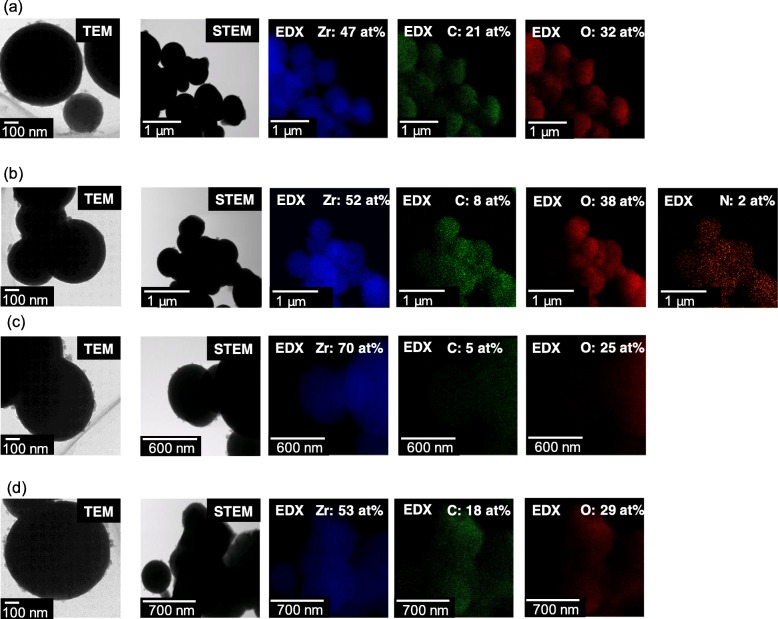
TEM and STEM-EDX mapping images of **a** HEMA-impregnated ZrO_2_ MARIMO, **b** NIPAM-impregnated ZrO_2_ MARIMO, **c** BMA-impregnated ZrO_2_ MARIMO, and **d** CHMA-impregnated ZrO_2_ MARIMO

To complete the systematic study on the relationship between the nano-concave-convex surface of the ZrO_2_ MARIMO filler and polymer chains, we modified the ZrO_2_ MARIMO filler surface by polymer impregnation to mask the nano-concave-convex surface. Herein, we selected vinyl monomers such as HEMA, NIPAM, BMA, and CHMA to be impregnated into pores of ZrO_2_ MARIMO. Polymerization of the impregnated monomers in ZrO_2_ MARIMO was achieved by UV irradiation in the presence of a photoinitiator (HCPK). The progress of polymerization was checked by SEC of the supernatant of the impregnated ZrO_2_ MARIMO/chloroform dispersion (Table S4). All the samples had molecular weights of around 1000. On DRIFTS experiments, there is no significant peak indicating interaction between the polymer and ZrO_2_ MARIMO (Fig. S8). In this meaning, we studied SEM and STEM-EDX analysis to confirm impregnation. As shown in Fig. 4, the spherical MARIMO morphologies were retained even after the impregnation treatments. STEM-EDX analysis (Fig. 5) clearly shows that Zr, C, O, and (N) atoms were homogeneously distributed throughout the ZrO_2_ MARIMO fillers. These results indicate that the monomers were uniformly impregnated and polymerized in the nanocavities and pores of the MARIMO.

### Mechanical Tensile Testings of Polymer Composites

**Fig. 6:**
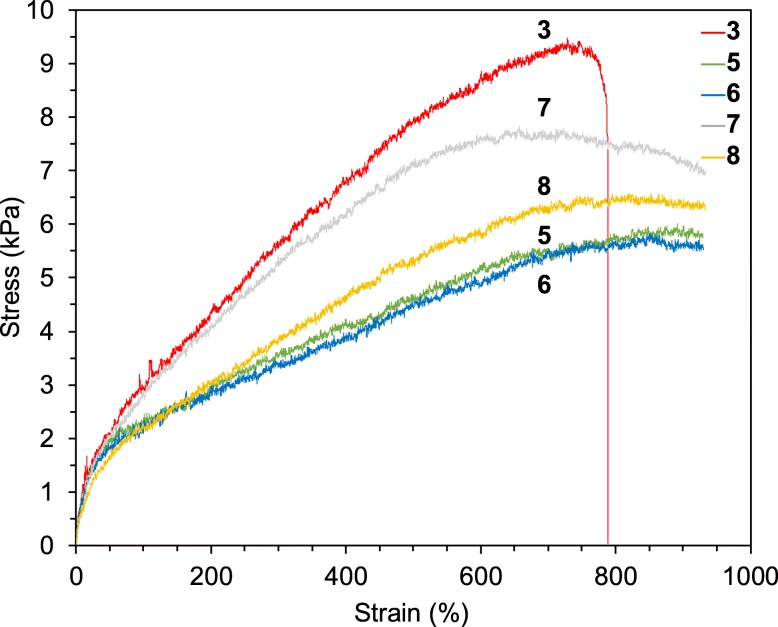
Tensile stress–strain curves for 30 wt% PNIPAM hydrogel composites with 0.02 wt% nano-concave-convex surface ZrO_2_ MARIMO (3), with 0.02 wt% HEMA-impregnated ZrO_2_ MARIMO (5), with 0.02 wt% NIPAM-impregnated ZrO_2_ MARIMO (6), with 0.02 wt% BMA-impregnated ZrO_2_ MARIMO (7), and with 0.02 wt% CHMA-impregnated ZrO_2_ MARIMO (8)

Existence of the anchoring effect of the nano-concave-convex surface of ZrO_2_ MARIMO was studied through mechanical tensile testings. Polymer composites with HEMA-impregnated ZrO_2_ MARIMO (5), with NIPAM-impregnated ZrO_2_ MARIMO (6), with BMA-impregnated ZrO_2_ MARIMO (7), and with CHMA-impregnated ZrO_2_ MARIMO (8) were prepared according to the similar procedures to that employed for composites 2b, 3, and 4. As shown by the mechanical tensile strengths obtained from stress–strain curves in Fig. 6, all composites 5–8 with vinyl polymer impregnated ZrO_2_ MARIMO fillers showed lower tensile strength as compared to composite 3 with the nano-concave-convex ZrO_2_ MARIMO filler (Table 3), suggesting the anchoring effect of the surface reduced in all cases of the polymer impregnated ZrO_2_ MARIMO fillers. Instead, higher elongation of all composites 5–8 with the polymer impregnated ZrO_2_ MARIMO fillers was clearly observed, which can be ascribed to slippage of the matrix polymer chains on the polymer-impregnated MARIMO filler surfaces. Thus, polymer-impregnated ZrO_2_ MARIMO fillers in polymer composites induced lower tensile strength and improved elongation capacity of the composites. Turning to the protopype ZrO_2_ MARIMO, the nano-concave-convex surface played a positive role in the tensile strength of the polymer composites.

**Table 3:** Tensile strengths and elongation capacities of 30 wt% PNIPAM hydrogel composites with 0.02 wt% polymer-impregnated ZrO_2_ MARIMOs

Composite	Filler	Stress_MAX_ (kPa)	Strain_MAX_ (%)
5	HEMA-impregnated ZrO_2_ MARIMO	6.1 ± 0.2	> 930^a^
6	NIPAM-impregnated ZrO_2_ MARIMO	5.9 ± 0.3	> 930^a^
7	BMA-impregnated ZrO_2_ MARIMO	7.9 ± 0.3	> 930^a^
8	CHCA-impregnated ZrO_2_ MARIMO	6.7 ± 0.2	> 930^a^

^a^The percent elongation more than 930% means the limitation of tensile tester machine

## Conclusion

Surface modification of a ZrO_2_ MARIMO filler with a nano-concave-convex structure revealed importance of nanoscale anchoring interactions between the filler surface and matrix polymer chains via mechanical tensile testings. To investigate the effect of the nano-concave-convex structure, we modified the ZrO_2_ MARIMO filler surface by (i) calcination of the ZrO_2_ MARIMO to smooth the nano-concave-convex surface and (ii) impregnation of polymers into the ZrO_2_ MARIMO pores to mask the nano-concave-convex surface. Mechanical tensile testing was applied to estimate the interaction between the surface of the fillers and the polymer chains in the polymer composites. The polymer composites containing a nano-concave-convex ZrO_2_ MARIMO filler showed the highest tensile strength, while polymer-impregnating the ZrO_2_ MARIMO fillers caused the large elongation. Thus, the nano-concave-convex surface of the ZrO_2_ MARIMO filler positively interacted with the matrix polymer chains to improve the tensile strength capacity, while polymer-masking the nano-concave-convex surface of the ZrO_2_ MARIMO fillers improved the elongation capacity. Consequently, rational design of the filler surface enabled us to understand the nanoscale interaction of filler surface with the polymer matrix through macro-scale mechanical tensile testings. Different kinds of monomers or polymers, such as ionic, hydrophilic, and hydrophobic monomers or polymers, can be incorporated into the MARIMO fillers by the simple impregnation technique to control properties of MARIMO fillers. Further studies on better dispersion of ZrO_2_ filler into aqueous media is now in progress.

## Supplementary information

**Additional file 1.** Supporting Information. Photographs of PNIPAM hydrogels, stress-strain curves and mechanical properties of polymer hydrogels, and SEC results of polymer impregnated ZrO_2_ MARIMOs.

## Data Availability

All relevant data that support the findings of this study are available from the corresponding author on request.

## Abbreviations

BET: Brunauer–Emmett–Teller
BF: Bright-field
BJH: Barrett–Joyner–Halenda
BMA: Benzyl methacrylate
CHMA: Cyclohexyl methacrylate
DRIFTS: Diffuse reflectance infrared Fourier transform spectroscopy
DSC: Differential scanning calorimetry
HCPK: 1-Hydroxycyclohexyl phenyl ketone
HEMA: 2-Hydroxyethyl methacrylate
KPS: Potassium persulfate
MARIMO: Micro/mesoporously architected roundly integrated metal oxide
NIPAM: *N*-Isopropylacrylamide
PNIPAM: Poly(*N*-isopropylacrylamide)
SEC: Size exclusion chromatography
SEM: Scanning electron microscopy
TEM: Transmission electron microscopy
TMEDA: *N,N,N',N'*-tetramethylethylenediamine
XRD: X-ray diffractometry
ZrO_2_: Zirconia
