# Supplementary material for: Nanoscale Effect of Zirconia Filler Surface on Mechanical Tensile Strength of Polymer Composites
Source: Nanoscale Res Lett. 2020 Mar 2;15:51. doi: 10.1186/s11671-020-3282-6 (PMC7052101; doi:10.1186/s11671-020-3282-6)
Supplement: Supplementary file 1 — Additional file 1. Supporting Information. Photographs of PNIPAM hydrogels, stress-strain curves and mechanical properties of polymer hydrogels, and SEC results of polymer impregnated ZrO2 MARIMOs. [file 11671_2020_3282_MOESM1_ESM.docx]

**Supporting Information**

**Nanoscale Effect of Zirconia Filler Surface on Mechanical Tensile Strength of Polymer Composites**

Kai Kan^1,2,3^, Daiki Moritoh^1^, Yuri Matsumoto^1^, Kanami Masuda^1^, Masataka Ohtani^1,2,3*^, Kazuya Kobiro^1,2,3*^

^1^School of Environmental Science and Engineering, Kochi University of Technology, 185 Miyanokuchi, Tosayamada, Kochi 782-8502, Japan

^2^Laboratory for Structural Nanochemistry, Kochi University of Technology, 185 Miyanokuchi, Tosayamada, Kochi 782-8502, Japan

^3^Research Center for Material Science and Engineering, Kochi University of Technology, 185 Miyanokuchi, Tosayamada, Kochi 782-8502, Japan

Corresponding Author (E-mail)

*E-mail: ohtani.masataka@kochi-tech.ac.jp

*E-mail: kobiro.kazuya@kochi-tech.ac.jp

**
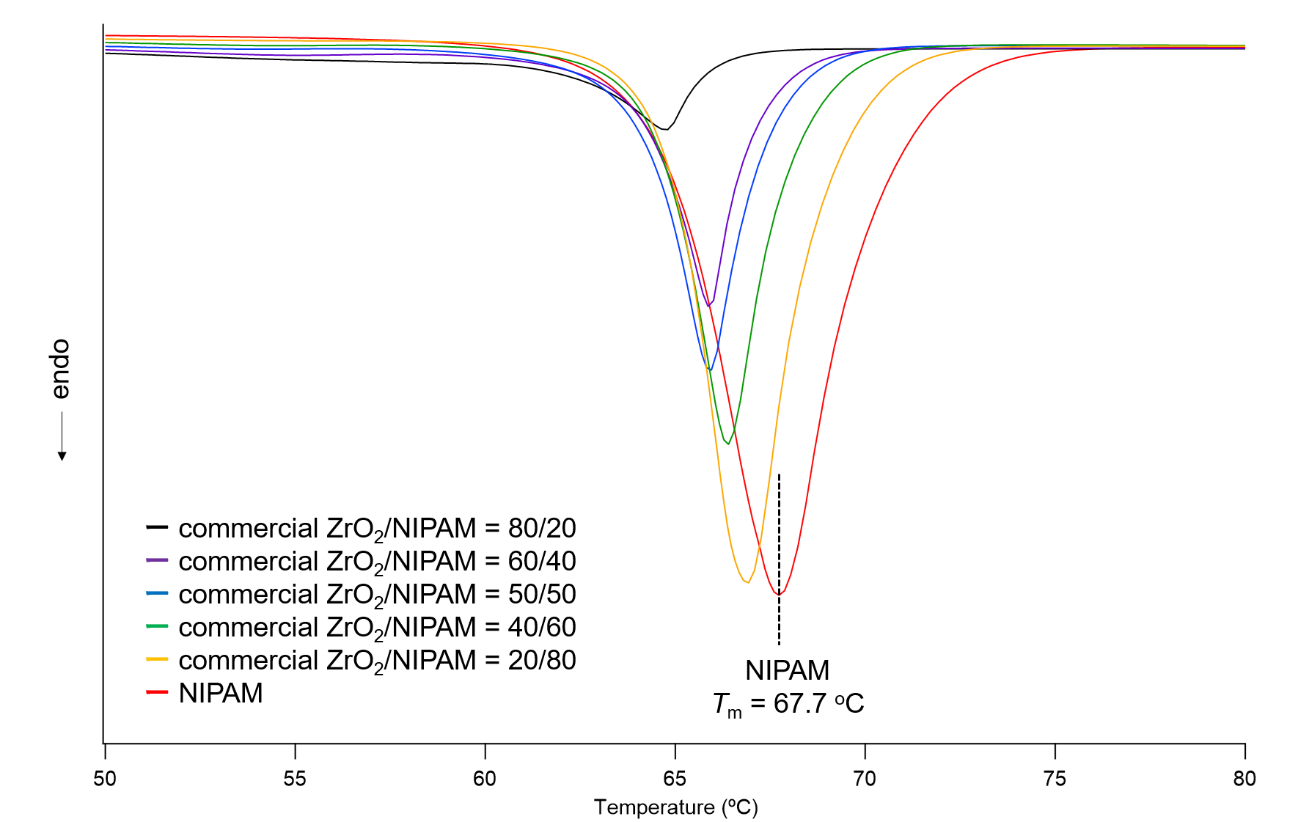
Fig. S1.** DSC profiles of commercial ZrO_2_ and NIPAM monomer mixtures with different ratio in weight % at the third heating scans.

**
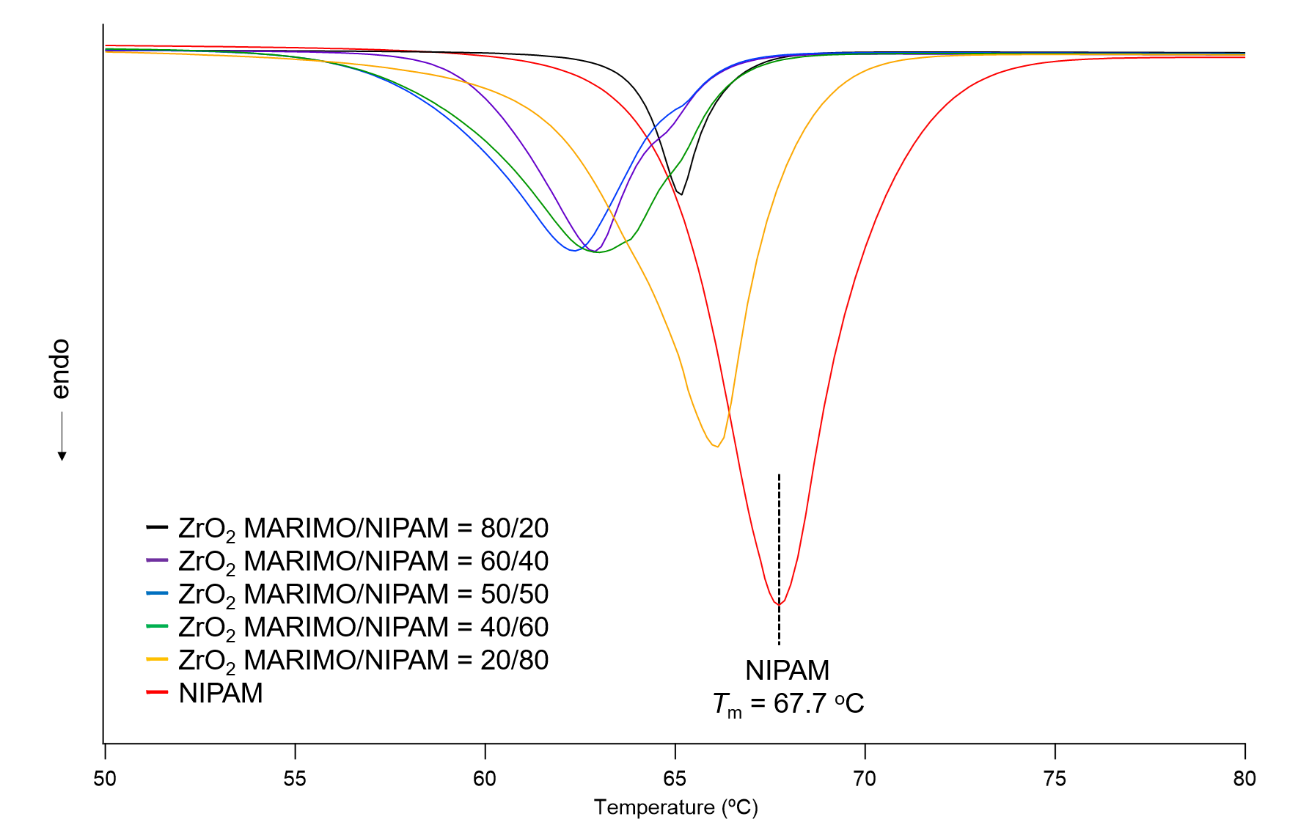
**

**Fig. S2.** DSC profiles of ZrO_2_ MARIMO and NIPAM monomer mixtures with different ratio in weight % at the third heating scans.

**
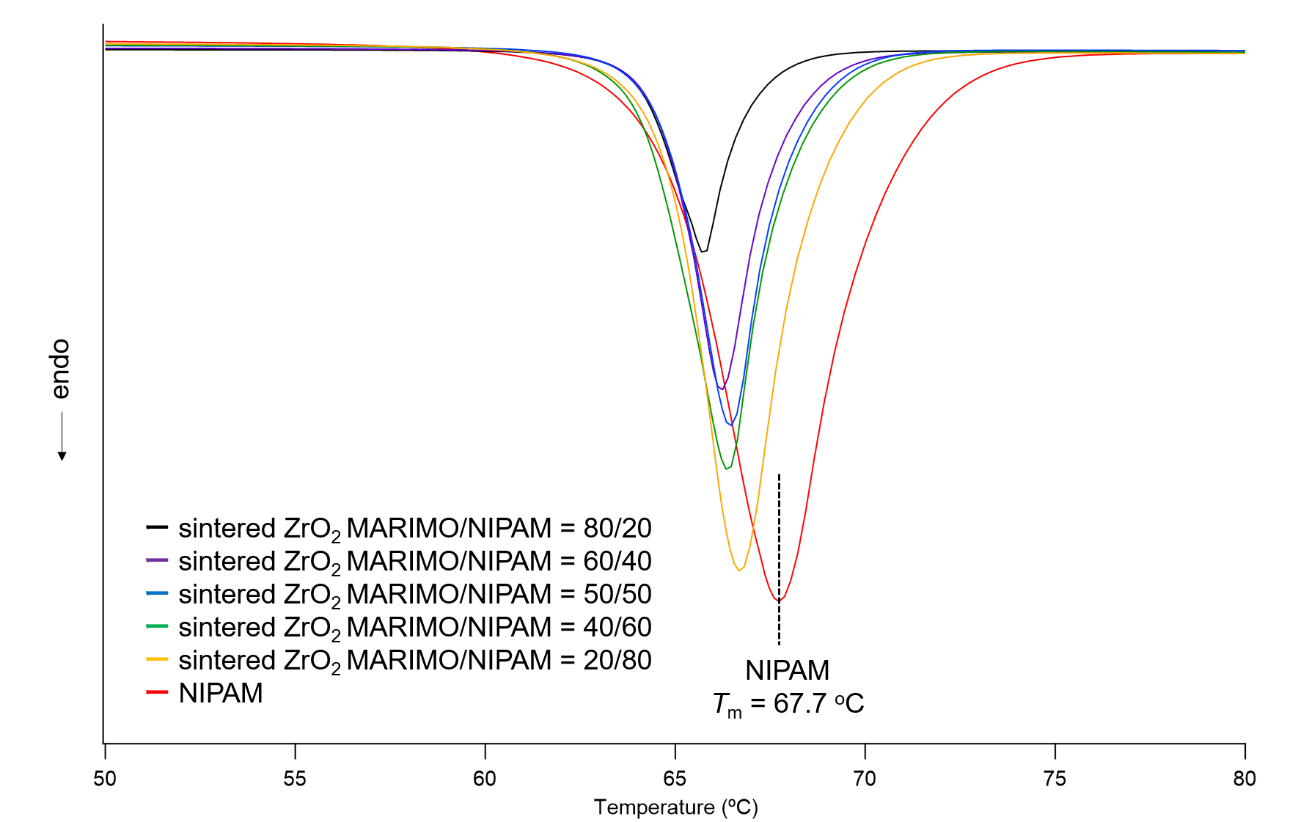
Fig. S3.** DSC profiles of sintered ZrO_2_ MARIMO and NIPAM monomer mixtures with different ratio in weight % at the third heating scans.

**Table S1.** Endothermal peaks of ZrO_2_ filler and NIPAM mixtures on DSC analysis

| Ratio (wt%) | Commercial ZrO_2_  *T*_m_ (^o^C) | ZrO_2_ MARIMO*^a^*  *T*_m_ (^o^C) | Sintered ZrO_2_ MARIMO *T*_m_ (^o^C) |
| --- | --- | --- | --- |
| 80/20 | 64.8 | 65.2 | 65.7 |
| 60/40 | 65.9 | 62.9 (65) | 66.2 |
| 50/50 | 66.0 | 62.4 (65) | 66.5 |
| 40/60 | 66.4 | 63.0 (65) | 66.3 |
| 20/80 | 66.9 | 66.1 | 66.7 |
| *^a^*An approximate position of shoulder was given in the parentheses. | | | |

**Fig. S4.** Photographs of PNIPAM hydrogels with (a) 20 wt%, (b) 25 wt%, and (c) 30 wt%, immediately after preparation and after 1 hour.

**Fig. S5.** Tensile stress–strain curves for 25 wt% and 30 wt% PNIPAM hydrogels.

**Table S2.** Tensile strengths and elongation capacities of 25 wt% and 30 wt% PNIPAM hydrogels

| Monomer ratio (wt%) | Stress_MAX_ (kPa) | Strain_MAX_ (%) |
| --- | --- | --- |
| 25 | 2.7 ± 0.2 | >930*^a^* |
| 30 | 7.8 ± 0.2 | 716 ± 106 |

*^a^* The percent elongation more than 930% means the limitation of tensile tester machine.

**Fig. S6.** Tensile stress–strain curves for 30 wt% PNIPAM hydrogels with 0.002 wt% (**2a**), 0.02 wt% (**2b**), and 0.04 wt% (**2c**) of commercial ZrO_2_ filler.

**Table S3.** Tensile strengths, elongation capacities, and work of 30 wt% PNIPAM hydrogels composites with different amounts of commercial ZrO_2_

| Composite | Ratio (wt%) | Stress_MAX_ (kPa) | Strain_MAX_ (%) | Work |
| --- | --- | --- | --- | --- |
| **2a** | 0.002 | 9.5 ± 0.7 | 525 ± 60 | 3783 ± 437 |
| **2b** | 0.02 | 7.7 ± 0.2 | 902 ± 28 | 5242 ± 246 |
| **2c** | 0.04 | 7.9 ± 0.7 | 695 ± 25 | 4193 ± 483 |

**Table S4.** Degree of polymerization of polymer-impregnated ZrO_2_ MARIMOs determined by SEC with polystyrene standard in chloroform at 40 °C

| Polymer-impregnated ZrO_2_ MARIMO | *M*_n_ ×10^3^ | *M*_w_ ×10^3^ | *M*_w_/*M*_n_ |
| --- | --- | --- | --- |
| HEMA | 1.3 | 1.6 | 1.2 |
| BMA | 1.1 | 1.3 | 1.1 |
| CHMA | 1.1 | 1.3 | 1.1 |

**Fig. S7.** SEM images of freeze-dried 30 wt% PNIPAM hydrogel composites with 0.02 wt% ZrO_2_ fillers. Composites with 0.02 wt% (a) commercial ZrO_2_, (b) nano-concave-convex ZrO_2_ MARIMO, and (c) sintered ZrO_2_ MARIMO.

**Fig. S8.** FT-IR spectra of NIPAM impregnated ZrO_2_ MARIMO, PNIPAM, and ZrO_2_ MARIMO measured in diffuse reflection mode.
